# Tau Causes Synapse Loss without Disrupting Calcium Homeostasis in the rTg4510 Model of Tauopathy

**DOI:** 10.1371/journal.pone.0080834

**Published:** 2013-11-20

**Authors:** Katherine J. Kopeikina, Susanne Wegmann, Rose Pitstick, George A. Carlson, Brian J. Bacskai, Rebecca A. Betensky, Bradley T. Hyman, Tara L. Spires-Jones

**Affiliations:** 1 Department of Anatomy and Neurobiology, Boston University School of Medicine, Boston, Massachusetts, United States of America; 2 MassGeneral Institute for Neurodegenerative Disease at Massachusetts General Hospital, Charlestown, Massachusetts, United States of America; 3 Harvard Medical School, Boston, Massachusetts, United States of America; 4 McLaughlin Res. Inst., Great Falls, Montana, United States of America; 5 Harvard School of Public Health, Boston, Massachusetts, United States of America; 6 Centre for Cognitive and Neural Systems, University of Edinburgh, Edinburgh, United Kingdom; University of South Florida Alzheimer's Institute, United States of America

## Abstract

Neurofibrillary tangles (NFTs) of tau are one of the defining hallmarks of Alzheimer’s disease (AD), and are closely associated with neuronal degeneration. Although it has been suggested that calcium dysregulation is important to AD pathogenesis, few studies have probed the link between calcium homeostasis, synapse loss and pathological changes in tau. Here we test the hypothesis that pathological changes in tau are associated with changes in calcium by utilizing in vivo calcium imaging in adult rTg4510 mice that exhibit severe tau pathology due to over-expression of human mutant P301L tau. We observe prominent dendritic spine loss without disruptions in calcium homeostasis, indicating that tangles do not disrupt this fundamental feature of neuronal health, and that tau likely induces spine loss in a calcium-independent manner.

## Introduction

The Alzheimer Disease (AD) brain accumulates intracellular neurofibrillary tangles, composed primarily of the microtubule associated protein tau and extracellular amyloid-β plaques. The AD brain is also characterized by synaptic and neuronal loss, which more closely correlate with cognitive decline in AD than the histological hallmarks of the disease [1-5]. Calcium dysregulation has been proposed to subserve synapse loss and is thought to be one of the earliest events in AD [6-10]. It is well known that mutations in genes that influence amyloid beta processing occur in familial variants of AD, and much emphasis has thus been placed in understanding the toxic consequences of Aβ, which has been linked to disruptions in calcium homeostasis in several models [11,12]. However, the role of calcium interactions in tau related AD pathogenesis and, in particular, in synapse loss has not been clearly delineated.

Calcium is a tightly regulated signaling molecule imperative to normal neuronal function. At the synapse, calcium is critical for inter-neuronal signaling, which underlies the processes involved in learning and memory [13,14]. At non-synaptic sites, calcium is critical for intra-neuronal signaling cascades, which when altered can lead to initiation of apoptotic cell death. Pathological increases in intracellular calcium levels have been demonstrated to increase levels of tau hyperphosphorylation [15,16], and tau accumulation in dendrites has been associated with local calcium elevations after application of Aβ to primary neurons in culture [12], leading to the supposition that tau alterations are downstream of calcium elevations. There is also some evidence for the inverse relationship, i.e. that pathological changes in tau are upstream of calcium dyshomeostasis. In cells that are exposed to extracellular tau or over-express tau, hyperphosphorylation, misfolding and mislocalization of tau is accompanied by disruptions in mitochondrial calcium buffering and cellular calcium homeostasis [8,12,17,18]. However, there are no data to our knowledge that test whether tau alterations are upstream of calcium dysregulation in the intact brain.

Traditionally, the tau protein has been known as a predominantly axonal protein, where it stabilizes microtubules and plays an important role in neuronal transport and function [19,20]. Recent studies indicate that tau may have additional functions beyond the axon, in dendrites and at post-synaptic sites [21-24]. These recent studies show that 1) tau plays a physiological role at dendritic spines [24], 2) tau and Aβ both cause synapse loss independently [25,26], and 3) elevated calcium levels are associated with Aβ–induced synapse loss [27,28], which leads to the question of whether the molecular cascade leading to tau induced synapse loss also involves increased calcium levels in dendrites and dendritic spines.

In this study, we used in vivo multiphoton imaging of yellow cameleon 3.6 (YC3.6), a ratiometric FRET based calcium indicator encoded and delivered by an adeno-associated virus (YC3.6AAV2) [27,29] to visualize intracellular calcium concentration in the brain of living adult mice that over-express human mutant P301L tau (rTg4510). At 8-9 months of age, these mice are beginning to lose neurons and to accumulate neurofibrillary tangles in the neocortex [30,31]. Further, P301L tau expressing neurons at this age have been shown to have either “atrophic” or “intact” morphologies with atrophic neurons demonstrating dendritic spine loss and loss of dendritic branches [32], indicating that this is a time when synapse loss is actively occurring. Here we demonstrate that 8-9 month old rTg4510 mice exhibit significant loss of dendritic spines but maintain baseline calcium levels in dendrites and dendritic spines despite accumulation of tau pathology. These data indicate that pathological changes in tau cause synapse loss in a calcium-independent manner, suggesting that pathological changes in tau may be downstream of both Aβ and calcium dysregulation in synapse loss in AD.

## Materials and Methods

### Animals

In this study, we used the rTg4510 mouse model of regulatable P301L human mutant tau over-expression. Mice were generated as previously described with a responder transgene containing a tetracycline-operon-responsive element (TRE) upstream of cDNA of full length, four-repeat (0N4R), P301L human tau and an activator transgene containing a tet-off open reading frame downstream of calcium calmodulin kinase II promoter elements [30,31,33]. When both activator and responder transgene are present, this bigenic model results in over-expression of human P301L tau limited to forebrain structures. Littermate animals containing only the activator transgene, which therefore do not express human mutant tau were used as controls. Animals were aged to 8-9 months before experiments began (n=9 rTg4510 and 6 controls). Animal experiments were conducted in accordance with NIH and Massachusetts General Hospital policies and were approved by the Massachusetts General Hospital Subcommittee on Research Animal Care.

### Yellow cameleon 3.6 AAV2

For in vivo calcium imaging, adeno-associated virus (AAV2) encoding the ratiometric calcium indicator yellow cameleon 3.6 (YC3.6) under the CBA promoter (YC3.6AAV2), was intracortically injected into rTg4510 and control mice as described previously [27,29]. The YC3.6AAV2 construct contains a fluorescence resonance energy transfer (FRET) based calcium indicator in which calcium calmodulin, a calcium binding peptide, is flanked by cyan and yellow fluorescent proteins (CFP and YFP respectively). Increases in calcium binding alter the conformation of the indicator, decrease the spatial distance between CFP and YFP, and increase FRET efficiency, resulting in an increase in the ratio (R) of YFP/CFP fluorescence. These ratios can be converted to calcium concentrations after all data have been collected using the equation [Ca^2+^]=K_d_*((R-R_min_)/(R_max_-R))^(1/Hill) where K_d_=277, Hill=1.1, R=ratio, R_min_=minimum ratio=1.36, R_max_=maximum ratio=2.48. Minimum and maximum ratios were defined as the median of the lowest 5% and highest 5% of the YC ratios for control animals. The viral titer was 5.0 x 10^12^.

### Animal surgeries

Intracortical injections and cranial window implantations were performed as previously described [27,34-36]. For intracortical injections, mice were anaesthetized with ketamine/xylazine (100 mg/kg, 10 mg/kg i.p.) and placed in a stereotax. Skin on the top of the head was sterilized, local anesthetic injected and a 2-3 mm incision made along the midline between the ears. Using a high-speed drill, 2 burr holes were drilled in the skull, 1.5 mm lateral to bregma bilaterally, 0.5 mm caudal on the left and 1.5 mm caudal on the right. 1.5 μL of YC3.6AAV2 was injected at a rate of 0.25 μL/min and at a depth of 0.8-1.2 mm into somatosensory cortex. Following one injection in each burr hole, the scalp was sutured and the mouse allowed to recover from anesthesia.

Following a three to four week incubation period to allow for YC3.6 expression, mice were anaesthetized with isoflurane and secured in a stereotax. The skin on the top of the head was sterilized, a local anesthetic injected, and a circle of skin covering the top of the head removed. A high-speed drill was used to make a 6 mm circular craniotomy centered over both injection sites. The brain was rinsed with PBS and an 8 mm sterilized glass coverslip placed over the exposed brain and glued to the skull with a Krazy Glue-dental cement mixture. Mice were given three weeks recovery to prevent any calcium artifacts due to swelling resultant from surgery. 

### Multiphoton in-vivo imaging

In vivo multiphoton images of YC3.6-filled neuronal processes and dendritic spines were obtained as described previously [27]. In short, mice were anaesthetized with isoflurane and placed in a stereotax within the stage of an Olympus BX61WI upright microscope with Olympus Fluoview 1000MPE, pre-chirp optics and fast AOM. A low melting point wax restraining ring was applied to the periphery of the coverslip and filled with distilled water to provide a well for the Olympus 20x 0.95 NA water immersion objective. A MaiTai titanium/sapphire laser (Spectra-Physics, Fremont CA) generated two-photon fluorescence at an excitation wavelength of 860 nm (laser power < 50 mW). Emitted light in the range of 380-480 nm (CFP) and 500-540 nm (YFP) was collected by photomultiplier tube detectors (Hamamatsu, Ichinocho, Japan). For dendrite analysis, image stacks were recorded at 0.8-1 μm z-step size, 50-80x magnification, 512x512 px, and a scan rate of 4-8 μs/pixel. Image stacks for spine analysis were collected at higher magnification (80-100x) and a step size of 0.8 μm. 

### Image analysis

Images were viewed and processed with ImageJ (National Institutes of Health open software) and analyzed without knowledge of condition. A custom-made macro was used to generate a merged CFP-YFP image and a background subtracted ratiometric image (R = (YFP - Background)/(CFP - Background). Regions of interest (dendrites and dendritic spines) were selected from ~10 volumes per animal in the CFP-YFP merged image and applied to the ratiometric image and measured to obtain YFP/CFP ratios (YC ratios). Translation of ratio values to calcium concentrations occurred only after statistical analysis. 

Spine density was analyzed using NeuronStudio (Mt Sinai School of Medicine, Computational Neurobiology and Imaging Center) in combination with ImageJ. YC3.6-filled neurites >20 μm in length with at least three spines were selected for analysis. Approximately 5-10 dendrites were selected for each animal. Image stacks were opened in NeuronStudio and a median blur filter run. The dendrite segment and spines were selected semi-automatically and confirmed by comparison to the 3D stack open in ImageJ in order to ensure that spines counted connected to the dendritic shaft. Dendritic spine density was calculated as spines per micrometer along the dendritic shaft and compared to the calcium ratio of that parent dendrite.

### Statistical analyses

Normality of datasets was assessed with Shapiro-Wilks tests. To determine whether calcium levels were significantly elevated in rTg4510 brains, YC ratios of rTg4510 and control mice were compared applying a mixed effects logistic regression model. This model takes into account correlation among dendrites and dendritic spines within mice by including random effects for mice. Calcium overload thresholds were determined for dendrites (Thr_dend_= 2.14) and dendritic spines (Thr_spine_= 2.24) individually and set as the 95^th^ percentile of the respective YC ratios in control animals. Dendritic spine density was analyzed by taking the mean dendritic spine density for each animal and comparing the means of the rTg4510 and control groups with student’s t-tests. To determine the correlations between calcium concentration and spine density, all dendrites used in the dendritic spine quantification were analyzed with linear regression analysis and spearman’s rho correlation analysis in JMP software.

## Results

### Calcium levels in dendrites and dendritic spines of rTg4510 mice are not significantly different from control

**Figure 1:**
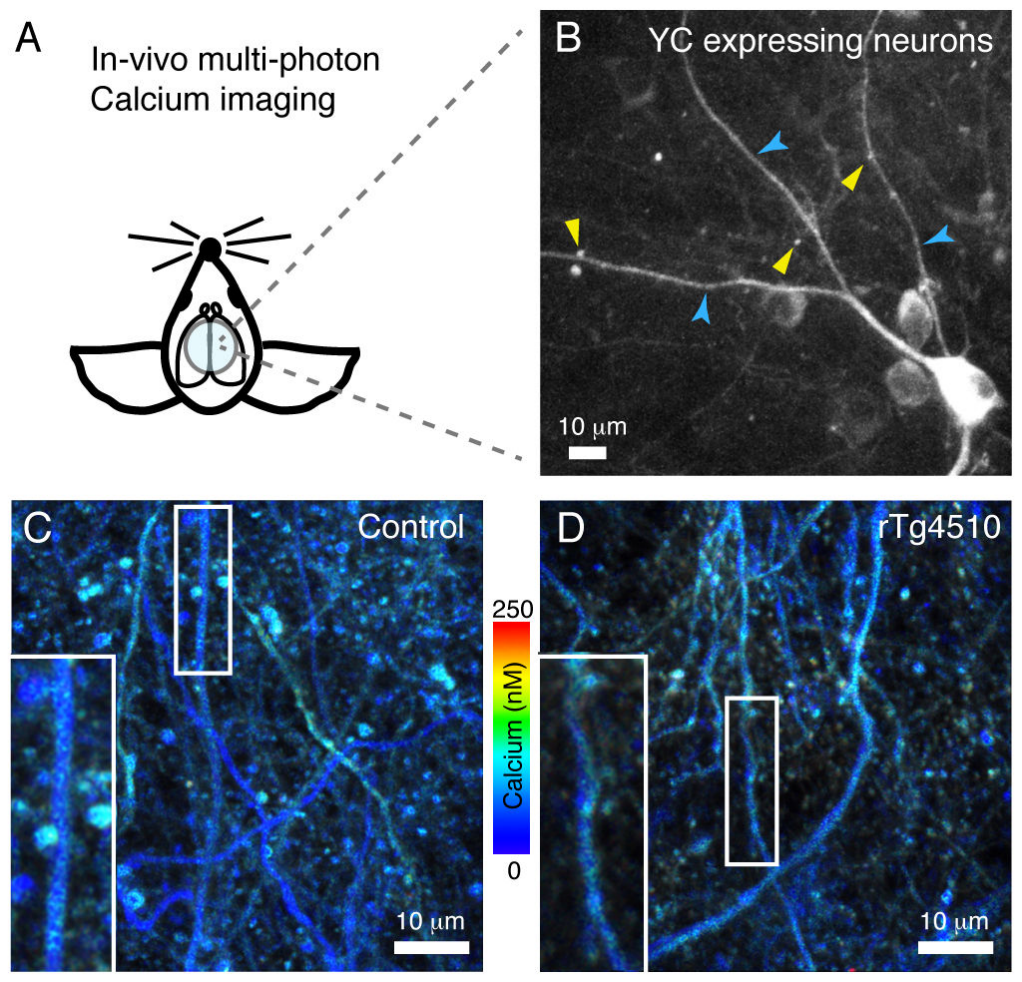
Yellow cameleon imaging in rTg4510 and control mice. In vivo multiphoton imaging of somatosensory cortex neurons expressing the ratiometric calcium indicator YC3.6 (A) demonstrates that the indicator fills both dendrites (B, blue arrow heads) and dendritic spines (B, yellow arrow heads). Calcium concentration images from control (C) and rTg4510 (D) mice were calculated from YC ratio (YFP/CFP) images and color-coded according to the color gradient shown in the middle. Scale bars (B, C, D) represent 10 μm.

**Figure 2:**
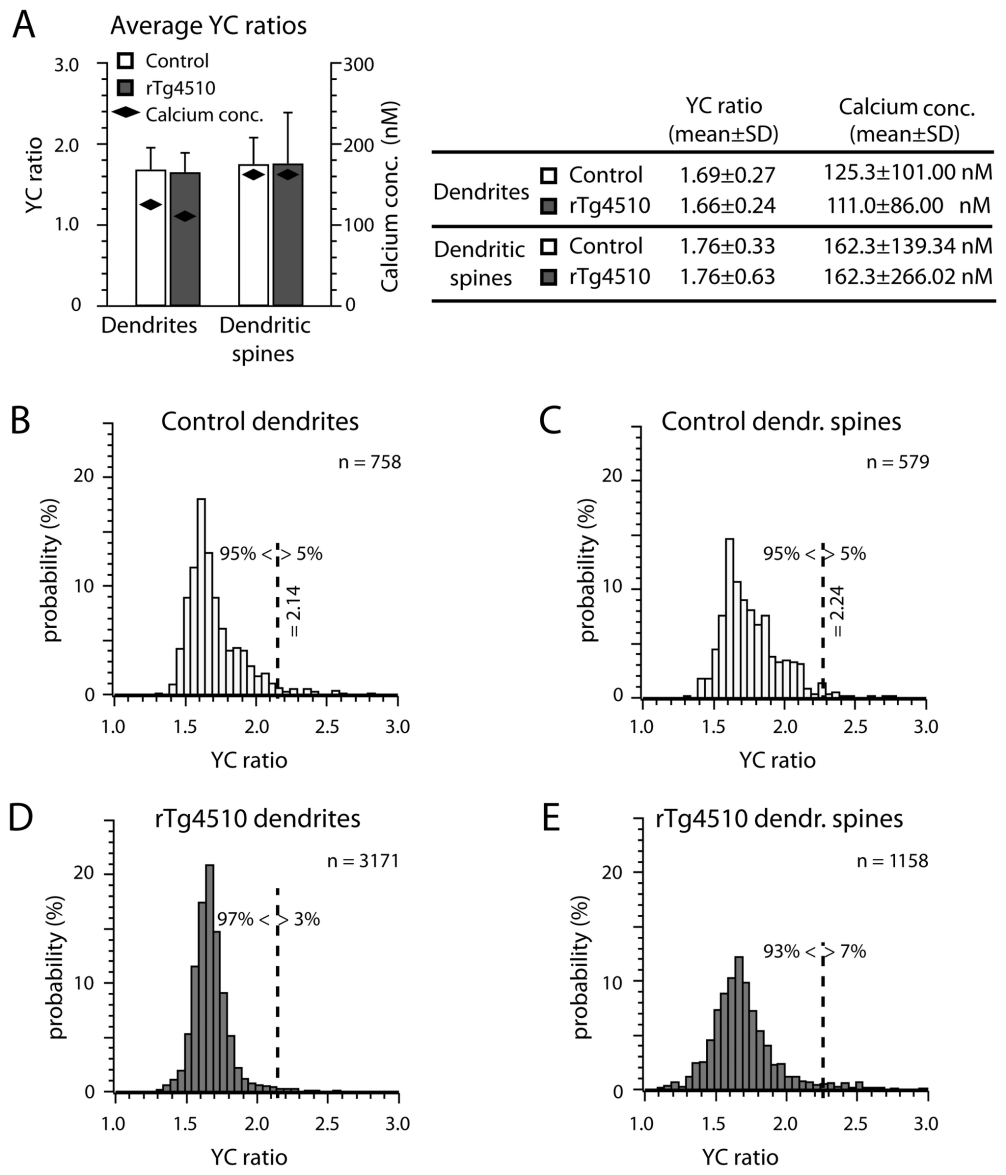
Calcium concentrations in dendrites and spines are not disrupted by tau over-expression. Average YC ratios (YFP/CFP) and calcium concentrations recorded from dendrites and dendritic spines in control and rTg4510 mice (A). Distributions of YC ratios in dendrites and dendritic spines from control (B, C) and rTg4510 mice (E, F) show no significant difference between control and rTg4510 mice. The dashed vertical lines indicate calcium overload thresholds at the 95^th^ percentile of the control mice data, determined for dendrites and spines separately. Data are shown as mean ± standard deviation.

Using in vivo multi-photon imaging of the ratiometric calcium indicator yellow cameleon (YC3.6) we quantified the resting calcium levels in neuronal processes of living 8-month old rTg4510 and control mouse brains. rTg4510 mice express human P301L tau in most cortical neurons (under the control of a tetracycline responsive element that is driven by a tet-off open reading frame downstream of CamKIIα promoter elements [30,37]) and at this age exhibit abnormal accumulation of tau in the soma of a subset of neurons (Figure S1). Injected YC3.6 fills cells, including dendrites, and dendritic spines (Figure 1). It has previously been demonstrated that changes in the YFP/CFP fluorescence ratio of the FRET-based YC3.6 can be utilized as a reliable readout to monitor intracellular calcium levels in the normal physiological range of neuronal calcium concentrations [27]. Analysis of 4655 dendrites across 15 mice (rTg4510 n=9, control n=6) demonstrated a maintenance of baseline calcium levels in rTg4510 mice, expressing high amounts of human P301L tau in the brain, when compared to control animals, with YC ratios of 1.69±0.27 (mean±SD) in control dendrites and 1.66±0.24 in rTg4510 dendrites, which translate into calcium concentrations of 125.3±101.00 nM and 111.0±86.00 nM respectively (Figure 2A, B, D). In plaque-bearing APP/PS1 mice, 20% of neurites demonstrated calcium overload [27]. To determine whether a similar subpopulation of neurites exhibit elevated calcium concentrations in rTg4510 mice, we identified the percentage of dendrites exceeding the YC ratio value for the 95^th^ percentile for control mice. Applying a mixed effects logistic regression model (Materials and Methods), no effect of tau overexpression on the number of dendrites and dendritic spines with overloaded calcium was detected (p>0.05). Similar to the data from dendrites, the average YC ratios and calcium concentrations in dendritic spines of rTg4510 (YC ratio 1.76±0.63, calcium 162.3±266.02 nM, n=1158 spines) were the same as in control mice and demonstrated no effect of tau over-expression on the number of spines exhibiting calcium overload (YC ratio 1.76±0.33, calcium 162.3±139.34 nM, Figure 2A, C, E).

### Spine loss in rTg4510 mice independent of calcium changes

**Figure 3:**
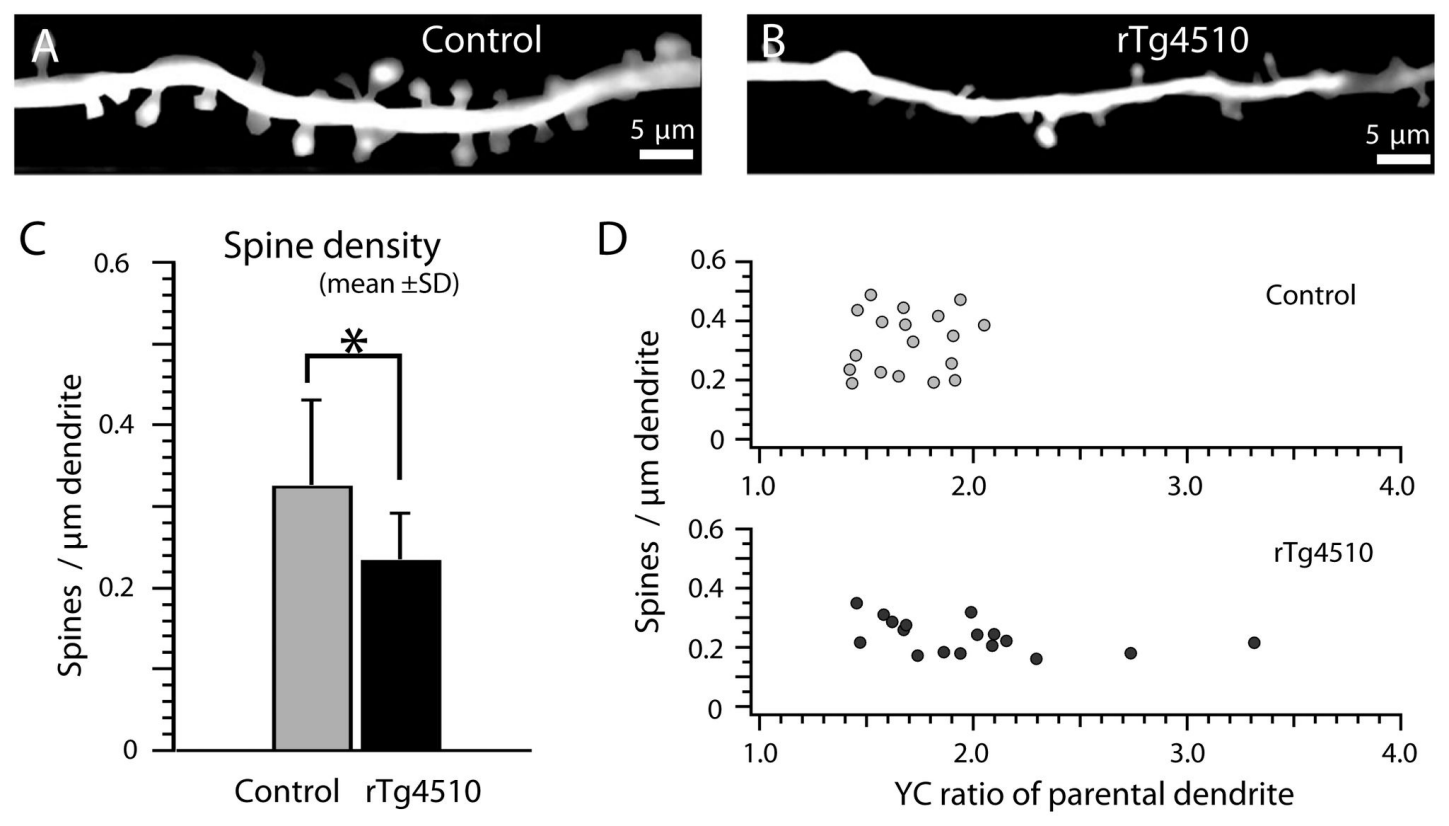
Spine loss in rTg4510 mice is independent of calcium dysregulation. High-resolution in vivo images of dendrites and spines from control (A) and rTg4510 (B) show substantial loss of spines in rTg4510 mice. For better visualization, the image background was removed and the contrast adjusted. Analysis of dendritic spine densities was performed on raw images. The average dendritic spine density (C) was significantly decreased in rTg4510 mice when compared to controls (*, p < 0.05). Spine density in rTg4510 mice did not significantly correlate with YC ratios in the parental dendrite (D). Scale bars (A, B) represent 5 μm.

 Studies of tau over-expressing neurons in culture have linked dendritic spine loss, the leading correlate to cognitive decline in AD [1,2,20], to alterations in calcium handling and mitochondrial mislocalization [12]. In addition, studies of Aβ toxicity have suggested that calcium dyshomeostasis precedes and leads to subsequent spine loss [8,9,14,16,38-42]. To determine whether calcium concentrations in the rTg4510 mice correlated with dendritic spine loss, spine density was calculated in rTg4510 and control mice and matched with the YC ratio of the parent dendrite in a subset of the dendrites imaged. At 8-9 months of age, with expression of human mutant P301L tau and tangles and neuronal loss in the somatosensory cortex, rTg4510 dendrites showed a nearly 30% decrease in spine density (p=0.003) as shown in Figure 3. This dramatic decrease in spine density in the rTg4510 brain did not correlate with the YC ratio of the parent dendrite (R^2^=0.197, multivariate pairwise correlation p=0.08, Figure 3), suggesting that tau induced synapse loss is not associated with sustained elevated calcium levels. We cannot exclude the possibility that YC ratios and spine density did at some point correlate, prior to such dramatic progression of pathology.

## Discussion

Many studies have demonstrated that alterations in APP processing and Aβ can induce calcium dysregulation and have established a strong link between increased calcium levels and dendritic spine loss [8,9,14,27,39,41,43,44]. However, the relationship between pathological changes in tau and calcium homeostasis [45,46] has rarely been addressed. Here we tested the hypothesis that the over-expression of human mutant P301L tau, which leads to tau deposition and severe neuronal loss in rTg4510 mice, alters calcium homeostasis and causes synapse loss. At 8-9 months of age these mice have extensive tau phosphorylation, neurofibrillary tangle formation, neuronal loss in the neocortex [31]. As demonstrated both in this study and previously, there is also substantial loss of dendritic spines on cortical pyramidal neurons at this age [25,47], but whether this is mediated by changes in intracellular calcium concentrations had not previously been studied.

 In general, tau mediated toxicity is thought to be primarily resultant from disruptions of microtubule-dependent neuronal transport processes, particularly in the anterograde direction, proving especially disruptive to mitochondrial distribution [48-51]. Pathological changes in tau, such as hyperphosphorylation, mislocalization and misfolding are known to have impair mitochondrial distribution and function [52-54]. In cultured neurons, Aβ causes increased calcium levels and dendritic spine loss concomitant with dendritic accumulation of tau and disrupted mitochondrial distribution [12]; however, whether aberrant accumulation of tau and mitochondrial distribution deficits are “upstream” or “downstream” of increased calcium was previously unknown. Here we demonstrate in a mouse model of tauopathy that in the absence of Aβ pathology, overexpression of P301L mutant tau causes distinct dendritic spine loss but no detectable increase in calcium concentrations in dendrites or dendritic spines. From this study and previous data examining mitochondrial distribution, we propose that the synaptic loss induced by tau may be due to impaired axonal transport of important synaptic cargoes including mitochondria, but that this does not require increased calcium concentrations in dendrites.

 Our data provide the first in vivo exploration of the relationship between pathological changes in tau and calcium homeostasis. We demonstrate that pathological changes in tau are sufficient to cause synapse loss without detectable alterations in resting calcium, which suggest different mechanisms of synaptic toxicity of tau and of Aβ. 

## Supporting Information

Methods S1 **Detail Western blot protocols and immunofluorescence labeling of brain sections used in Figure S1.**
(DOCX)

Figure S1 **Expression of human P301L tau in rTg4510 cortex.** Western Blot analysis of cortical brain extracts from 8-month old mice shows substantial amounts of human tau, detected by the human tau specific antibody HT7, in rTg4510 but not in control mice (A). Human tau immunolabeling in paraformaldehyde fixed coronal sections of somatosensory cortex, this time using human tau specific antibody Tau13 (B), verifies strong cortical expression of human P301L tau in 9-month old rTg4510 mice. Scale bars (B) represent 50 μm.
(TIFF)
