# Supplementary material for: Tau Causes Synapse Loss without Disrupting Calcium Homeostasis in the rTg4510 Model of Tauopathy
Source: PLoS One. 2013 Nov 20;8(11):e80834. doi: 10.1371/journal.pone.0080834 (PMC3835324; doi:10.1371/journal.pone.0080834)
Supplement: Methods S1 — Detail Western blot protocols and immunofluorescence labeling of brain sections used in Figure S1. (DOCX) [file pone.0080834.s001.docx]

SUPPORTING INFORMATION

**Supplemental Methods**

*Western Blot Analyses of Cortical Cytosolic Fractions*

Mice were euthanized with CO_2_ and cortical tissue was dissected, snap frozen and stored at -80° C. For homogenization, 1.5 ml cold buffer (20 mM HEPES pH 7.5, 300 mM NaCl, 2 mM DTT) supplemented with protease (Roche) and phosphatase (Sigma) inhibitors were added to each dissected cortex. Cortices were thawed on ice, gently ground using a Potter-Elvehjem homogenizer, and then passed 2-times through nylon filters (pore size 80 μm, Millipore) to remove debris. Homogenates were centrifuged at 12,000 g for 15 min to remove cellular compartments and the supernatant was centrifuged again at 100,000 g for 1 hour to remove membranes and obtain the cytosolic fraction. Protein concentrations were determined after addition of 1.5% SDS and boiling for 5 min by BCA assay (Pierce).

For SDS-PAGE, lysates (20 μg protein) were diluted in sample buffer containing reducing agent and lithium dodecyl sulfate (Invitrogen) and boiled again, loaded on NuPage Bis-Tris 4-12% gels, and transferred to nitrocellulose membranes. For Western Blot analyses, membranes were blocked in Odyssey Block buffer (Li-Cor Biosciences) and then incubated with primary antibodies (rabbit anti β-actin (Sigma, 1:10000) and mouse anti-human tau antibody HT7 (Thermo Scientific, 1:5000) overnight at 4° C. As secondary antibodies, anti-rabbit IgG conjugated to IRdye 680 and anti-mouse IgG conjugated to IRdye800 (Li-Cor Biosciences, 1:5000) were used and band intensities were recorded with an Odyssey infrared imager (Li-cor Bioscience).

Immunofluorescent Labeling of Coronal Brain Sections

rTg4510 and control mice were anaesthetized with isofluorane and perfused with 10 ml PBS followed by 10 ml 4%-paraformaldehyde in PBS. Brains were taken and drop fixed for two more days in 4%-paraformaldehyde in PBS, then transferred and stored in 0.25%-paraformaldehyde in PBS, and finally incubated for 2 days in 30% sucrose in PBS at 4° C for cryoprotection. For cutting of brain sections, sucrose protected brains were frozen onto a microtome stage, cut into 50 μm thick coronal sections, and somatosensory cortex sections were stored in 30% glycerol in PBS at -80° C.

To detect human tau, brain sections were rinsed briefly in PBS, permeabilized in 0.5% TX-100 in PBS for 30 min, washed 3-times in PBS, and blocked in PBS containing 5% normal goat serum (NGS) for 1 hour. For immunolabeling of human tau, sections were incubated in human tau specific primary antibody Tau13 (Abcam, 1:1000) diluted in PBS containing 5% NGS overnight at 4° C. After washing the sections 3-times in PBS, the secondary antibody (anti-mouse IgG conjugated to Cy3, Invitrogen, 1:1000) was applied for 1 hour at room temperature, sections were washed again and mounted onto microscope slides using DAPI containing mounting medium (Vectashield, VECTA). Imaging was performed using a 10x air objective on an epifluorescence microscope (ZEISS axio imager Z2).

**Supplemental Figure 1: Expression of human P301L tau in rTg4510 cortex.** Western Blot analysis of cortical brain extracts from 8-month old mice shows substantial amounts of human tau, detected by the human tau specific antibody HT7, in rTg4510 but not in control mice (A). Human tau immunolabeling in paraformaldehyde fixed coronal sections of somatosensory cortex, this time using human tau specific antibody Tau13 (B), verifies strong cortical expression of human P301L tau in 9-month old rTg4510 mice. Scale bars (B) represent 50 μm.
