# Supplementary figures and images for: Tau Causes Synapse Loss without Disrupting Calcium Homeostasis in the rTg4510 Model of Tauopathy
Source: PLoS One. 2013 Nov 20;8(11):e80834. doi: 10.1371/journal.pone.0080834 (PMC3835324; doi:10.1371/journal.pone.0080834)

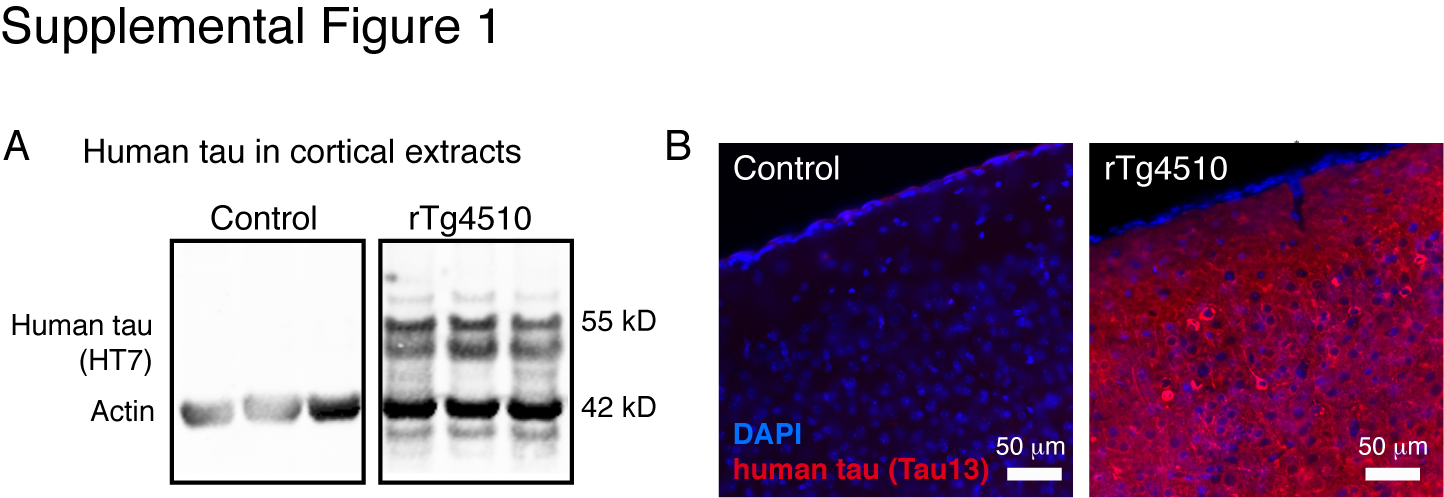

Supplement: Figure S1 — Expression of human P301L tau in rTg4510 cortex. Western Blot analysis of cortical brain extracts from 8-month old mice shows substantial amounts of human tau, detected by the human tau specific antibody HT7, in rTg4510 but not in control mice (A). Human tau immunolabeling in paraformaldehyde fixed coronal sections of somatosensory cortex, this time using human tau specific antibody Tau13 (B), verifies strong cortical expression of human P301L tau in 9-month old rTg4510 mice. Scale bars (B) represent 50 μm. (TIFF) [file pone.0080834.s002.tiff]
